# Association Between Rates of Down Syndrome Diagnosis in States With vs Without 20-Week Abortion Bans From 2011 to 2018

**DOI:** 10.1001/jamanetworkopen.2023.3684

**Published:** 2023-03-21

**Authors:** Sarina R. Chaiken, Ava D. Mandelbaum, Bharti Garg, Uma Doshi, Claire H. Packer, Aaron B. Caughey

**Affiliations:** 1 Department of Obstetrics and Gynecology, Oregon Health & Science University, Portland; 2 Department of Obstetrics and Gynecology, Brigham and Women’s Hospital, Boston, Massachusetts

## Abstract

**Importance:**

Many states enacted 20-week abortion bans from 2011 to 2018. Such bans affect individuals who receive diagnoses of fetal anomalies and aneuploidy in the second trimester, preventing pregnant individuals from having the choice of whether or not to continue the pregnancy.

**Objectives:**

To examine the trends of neonatal Down syndrome rates and assess the association between enactment of 20-week abortion bans and rates of Down syndrome diagnosis.

**Design, Setting, and Participants:**

This population-based, historical cohort study used National Vital Statistics System data on 31 157 506 births in the US from 2011 to 2018. Statistical analysis was performed from May 2021 to February 2023.

**Exposure:**

States were categorized as those with or without a 20-week abortion ban enacted during the study period.

**Main Outcomes and Measures:**

Demographic characteristics between the ban and no-ban states were compared using χ^2^ tests and 2-sample *t* tests. Multivariable logistic regression evaluated the adjusted odds of Down syndrome among births in states that enacted 20-week abortion bans after the abortion ban enactment, adjusting for state, year of birth, maternal race and ethnicity, age, educational level, insurance, and number of prenatal visits.

**Results:**

The cohort consisted of 31 157 506 births (mean [SD] maternal age, 28.4 [5.9] years) in the United States, of whom 15 951 neonates (0.05%) received a diagnosis of Down syndrome at birth. A total of 17 states enacted 20-week abortion bans during the study period, and 33 states did not enact bans. In both states with and states without bans, the birth prevalence of neonatal Down syndrome increased over time; in states with bans, rates increased from 48.0 to 58.4 per 100 000 births; in states without bans, rates increased from 47.4 to 53.3 per 100 000 births. In multivariable logistic regression assessing the interaction of time and presence of a 20-week abortion ban, the odds of Down syndrome were higher in states that enacted 20-week abortion bans after enactment of the law compared with the years prior to enactment of the ban (adjusted odds ratio, 1.22; 95% CI, 1.11-1.35).

**Conclusions and Relevance:**

In the US from 2011 to 2018, neonatal Down syndrome diagnoses increased more in states that enacted 20-week abortion bans compared with states that did not enact bans. Because these abortion bans were enacted throughout the study period and are known to inhibit choice in patient decision-making, it is possible that the difference in the rates of diagnosis is associated with these policies.

## Key Points

**Question:**

Is there an association between rates of neonatal Down syndrome diagnosis in states that did vs did not enact 20-week abortion bans from 2011 to 2018?

**Findings:**

In this cohort study of 31 157 506 births in the United States from 2011 to 2018, the birth prevalence of Down syndrome at birth increased over time, with a significantly higher increase among births in states that enacted a 20-week ban.

**Meaning:**

This study suggests that abortion bans may inhibit choice in decision-making in the context of second-trimester aneuploidy screening.

## Teaser

This cohort study uses National Vital Statistics System data to examine the trends of neonatal Down syndrome rates and assess the association between enactment of 20-week abortion bans and rates of Down syndrome diagnosis.

## Introduction

Legislation restricting abortion access significantly increased from 2011 to 2018.^1,2,3,4,5^ Although the 1973 US Supreme Court *Roe v Wade* decision established the right to abortion until fetal viability, many states have since passed bans prohibiting abortion after specific points in pregnancy.^6,7,8^ In 2022, 18 states had implemented abortion bans after 20 weeks’ gestational age, and 1 state prohibits abortion beyond 6 weeks after fertilization.^1^ These bans were predominantly enacted between 2010 and 2018 in tandem with more than 500 additional abortion restrictions passed in a similar time frame.^5,9^ These laws have been associated with worse outcomes for individuals denied an abortion, including delays in care, longer travel times, higher costs, and increased birth complications.^10,11,12,13^ Furthermore, navigating barriers to obstetric care and coping with the undue burden of an unintended child may result in short- and long-term psychosocial stress for the pregnant individual and their families.^14,15,16^

Twenty-week abortion bans affect individuals’ ability to have an abortion after second-trimester testing, including for diagnoses of fetal chromosomal abnormalities such as trisomy 21 or Down syndrome.^3,17^ Although advances in earlier prenatal screening and genetic testing allow for screening prior to 20 weeks, many cases are often not definitively diagnosed until 20 weeks or beyond.^18,19^ In these cases, patients in states with 20-week abortion bans may have insufficient time to make an informed decision and may be denied desired abortion care. Down syndrome is the most common chromosomal disorder, affecting approximately 1 in every 700 births.^20^ A systematic review on published literature in the US has estimated that termination rates range from 67% to 85% among the overall population of individuals with a positive prenatal diagnosis of Down syndrome.^21^

There remains a paucity of literature regarding how the increase in 20-week abortion bans were associated with rates of neonatal Down syndrome. Evaluating the association of these bans with the rate of fetuses with Down syndrome carried to viability is necessary to optimize health planning and better estimate the burden on people facing further restrictions to abortion care, given recent legislature.^22^ Therefore, this study aimed to examine the association between rates of Down syndrome diagnosed in states with and states without 20-week abortion bans in the US from 2011 to 2018 to better understand how such bans were associated with outcomes in pregnancies with Down syndrome.

## Methods

**Figure 1.**
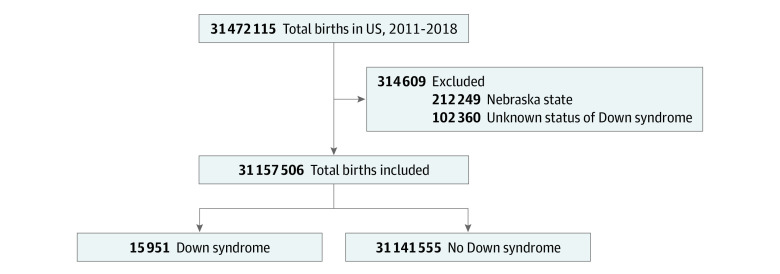
Inclusion and Exclusion Criteria Births in Nebraska were excluded because this state enacted a 20-week ban prior to 2011.

We performed a historical, population-based study using linked vital statistics–infant deaths data collected by the National Center of Health Statistics (2011-2018) to examine the association between enactment of 20-week abortion bans and neonates who received a diagnosis of Down syndrome. We excluded births with an unknown status of neonatal Down syndrome diagnosis (0.3% missing). In addition, births in Nebraska were also excluded because this state enacted a 20-week ban prior to 2011 (Figure 1). This study was considered exempt by the institutional review board at Oregon Health & Science University due to the deidentified nature of the vital statistics data set. Because these data were taken from a publicly available data set, no participant consent was required. This study followed the Strengthening the Reporting of Observational Studies in Epidemiology (STROBE) reporting guideline.

Our independent variable of interest was states that enacted 20-week abortion vs states that did not, which was based on data from the Guttmacher Institute.^23^ We included 17 states in the 20-week abortion ban group: Alabama, Arkansas, Georgia, Indiana, Iowa, Kansas, Kentucky, Louisiana, Mississippi, North Dakota, Ohio, Oklahoma, South Carolina, South Dakota, Texas, West Virginia, and Wisconsin. The enactment of 20-week abortion bans occurred in 2011 (Alabama, Indiana, Kansas, and Oklahoma), 2012 (Georgia and Louisiana), 2013 (Arkansas and North Dakota), 2014 (Mississippi), 2015 (Ohio, Texas, West Virginia, and Wisconsin), 2016 (South Carolina and South Dakota), and 2017 (Kentucky and Iowa). Thirty-two states, along with the District of Columbia (33 total), were included in the nonban group: Alaska, Arizona, California, Colorado, Connecticut, Delaware, Florida, Hawaii, Idaho, Illinois, Maine, Maryland, Massachusetts, Michigan, Minnesota, Missouri, Montana, Nevada, New Hampshire, New Jersey, New Mexico, New York, North Carolina, Oregon, Pennsylvania, Rhode Island, Tennessee, Utah, Vermont, Virginia, Washington, Washington DC, and Wyoming.

Our primary outcome of interest was neonatal Down syndrome diagnosis, which was captured using National Vital Statistics System data. Demographic characteristics were self-reported in the birth certificate data. Based on National Center of Health Statistics classifications, race and ethnicity were categorized into American Indian or Alaska Native, Asian, Black or African American, other or multiracial (including Native Hawaiian or Other Pacific Islander), and White. Race and ethnicity were included in this study as previous studies have shown that race and ethnicity may impact the sensitivity of Down syndrome diagnosis in birth certificate data.^24,25^ Maternal age was captured as a continuous variable. We categorized insurance (public or private), maternal educational level (some college or a college degree and higher), and number of prenatal visits (<5 visits and ≥5 visits).

### Statistical Analysis

Statistical analysis was performed from May 2021 to February 2023. All analyses were performed with an a priori significance threshold of *P* = .05, and hypothesis tests were 2-sided. Analyses were performed using Stata, version 17 (StataCorp LLC). χ^2^ Tests (categorical variables) and independent 2-sample *t* tests (continuous variables) were used to assess differences in demographic and individual characteristics among those in states with vs without 20-week abortion bans. We looked at the proportions of neonatal Down syndrome diagnoses over time (2011-2018) in 2 groups (states that enacted a 20-week abortion ban vs no ban) and presented the proportions as Down syndrome rates per 100 000 births. We graphically assessed the trends in Down syndrome diagnoses in both the ban and no-ban groups from 2011 to 2018.

Multivariable logistic regression analyses were then used to assess the association between the enactment of 20-week abortion bans and rates of Down syndrome diagnosis, after controlling for race and ethnicity, maternal age, insurance status educational level, number of prenatal visits, and year of birth. We also assessed this association in each year (2011-2018) using multivariable logistic regression models controlling for race and ethnicity, maternal age, insurance status, educational level, and number of prenatal visits.

To compare the rates of Down syndrome between states with and states without 20-week abortion bans after enactment of the law, we assessed the interaction of time (before abortion ban enactment vs the years of or after abortion ban enactment) and group (states with vs without 20-week abortion ban) in a multivariable logistic regression model. The regression model was a variation of the standard difference-in-difference model and controlled for state indicators, year of birth indicators, maternal race and ethnicity, maternal age, educational level, insurance status, and number of prenatal visits. This approach controls for secular trends and for unobserved state differences. It is a conservative approach when the standard difference-in-difference model cannot be used due to a violation in one of its assumptions. In our study, the preabortion ban–enactment trends in states with vs without a 20-week abortion ban were not parallel, violating this assumption of difference-in-difference models.

For the multivariable regression model controlling for year of ban enactment, states without a 20-week abortion ban were categorized at the median year of ban enactment (2014) to allow a comparison of states with vs states without abortion bans. Sensitivity analyses were performed using 2015 and 2016 as cutoffs for the states without bans as well.

## Results

We included 31 157 506 births (mean [SD] maternal age, 28.4 [5.9] years) that occurred in the United States from 2011 to 2018 (Figure 1). Of these, 33.1% were in states that enacted 20-week abortion bans during this period. Overall, 15 951 births (0.05%) were associated with a Down syndrome diagnosis, of which 5330 were in states that enacted 20-week abortion bans and 10 621 were in states without 20-week abortion bans (*P* = .36) (Table 1).

**Table 1.** Demographic and Clinical Characteristics Among States With or Without 20-Week Abortion Ban Enactment in United States From 2011 to 2018

Characteristic	% (95% CI)		*P* value^a^
	With 20-wk abortion ban (17 states)	No 20-wk abortion ban (33 states)	
Births, No.	10 305 246	20 852 260	NA
Maternal race and ethnicity			
American Indian or Alaska Native	0.86 (0.86-0.87)	0.82 (0.82-0.83)	<.001
Asian	3.40 (3.40-3.42)	7.67 (7.66-7.68)	
Black	17.5 (17.4-17.5)	12.89 (12.87-12.90)	
Hispanic	20.89 (20.86-20.91)	24.73 (24.71-24.75)	
White	55.66 (55.62-55.69)	51.37 (51.35-51.39)	
Other^b^	1.72 (1.71-1.72)	2.51 (2.51-2.52)	
Maternal age, mean (SD)	27.5 (5.8)	28.9 (5.9)	<.001
Public insurance	51.55 (51.51-51.58)	45.18 (45.16-45.20)	<.001
Less than college degree	65.33 (65.30-65.36)	59.02 (59.00-59.05)	<.001
Prenatal visits (<5)	6.46 (6.44-6.47)	4.54 (4.53-4.55)	<.001
Down syndrome	0.0517 (0.0503-0.0531)	0.0509 (0.0499-0.0519)	.36

Abbreviation: NA, not applicable.

^a^ χ^2^ Test or independent 2-sample *t* test used.

^b^ Other race or multiracial (including Native Hawaiian or Other Pacific Islander).

Demographic and individual characteristics of individuals giving birth differed between states with vs without 20-week abortion bans. In states that enacted 20-week abortion bans, a higher proportion of individuals giving birth were White (55.7% vs 51.4%; *P* < .001) or Black (17.5% vs 12.9%; *P* < .001), had public insurance (51.6% vs 45.2%; *P* < .001), had less than a college-level education (65.3% vs 59.0%; *P* < .001), and had fewer than 5 prenatal visits (6.5% vs 4.5%; *P* < .001) (Table 1).

**Figure 2.**
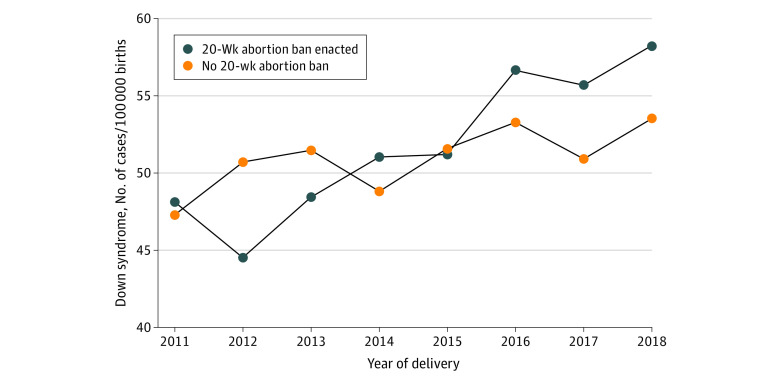
Rates of Neonatal Down Syndrome Diagnosis From 2011 to 2018 in States With 20-Week Abortion Bans and Those Without 20-Week Abortion Bans

From 2011 to 2018, rates of Down syndrome diagnosis in states that did not pass a 20-week abortion ban increased from 47.3 to 53.5 per 100 000 births, while among states that did enact a 20-week abortion ban, rates increased from 48.1 to 58.2 per 100 000 births (Table 2; Figure 2).

**Table 2.** Down Syndrome Diagnoses by Year Among States With 20-Week Abortion Bans and States Without 20-Week Abortion Bans^a^

	2011	2012	2013	2014	2015	2016	2017	2018	Overall
**No ban enacted**									
Down syndrome diagnosis, No. (cases per 100 000)	1243 (47.3)	1335 (50.7)	1346 (51.5)	1290 (48.7)	1360 (51.6)	1397 (53.3)	1303 (50.8)	1347 (53.5)	10 621 (50.9)
Total births, No.	2 628 369	2 630 976	2 613 008	2 646 353	2 635 842	2 619 913	2 562 535	2 515 264	20 852 260
**Ban enacted**									
Down syndrome diagnosis, No. (cases per 100 000)	613 (48.1)	572 (44.5)	623 (48.4)	670 (51.0)	676 (51.2)	738 (56.7)	708 (55.7)	730 (58.2)	5330 (51.7)
Total births, No.	1 273 898	1 284 975	1 286 338	1 312 524	1 319 797	1 302 315	1 271 092	1 254 307	10 305 246
Odds of Down syndrome vs states with no ban, aOR (95% CI)	1.19 (1.06-1.33)	0.99 (0.88-1.11)	1.08 (0.97-1.21)	1.14 (1.03-1.27)	1.10 (0.99-1.22)	1.21 (1.10-1.34)	1.32 (1.19-1.46)	1.33 (1.20-1.46)	1.17 (1.13-1.22)

Abbreviation: aOR, adjusted odds ratio.

^a^ Multivariable logistic regression showing odds of Down syndrome diagnosis in states that enacted 20-week abortion ban compared with states that did not enact a ban by year. Multivariable logistic regression for all deliveries controlled for race and ethnicity, maternal age, insurance status, educational level, number of prenatal visits, and year of birth. Additional multivariable regression for each year controlled for race and ethnicity, maternal age, insurance status, educational level, and number of prenatal visits.

Multivariable logistic regression analyses controlling for race and ethnicity, maternal age, insurance status, educational level, number of prenatal visits, and year of birth showed that, overall, deliveries in states that enacted a 20-week abortion ban were 1.17 times as likely (95% CI, 1.13-1.22) to have an associated neonatal diagnosis of Down syndrome compared with those in states without 20-week abortion bans (Table 2). On multivariable regression analyses by year of birth, there was a statistically significantly higher odds of Down syndrome diagnosis in states with abortion bans from 2015 to 2018, with increasing adjusted odds ratios each year. In 2015, deliveries in states that enacted bans were 1.10 times as likely (95% CI, 0.99-1.22) to have a diagnosis of Down syndrome, whereas, in 2018, they were 1.33 times as likely (95% CI, 1.20-1.46).

**Figure 3.**
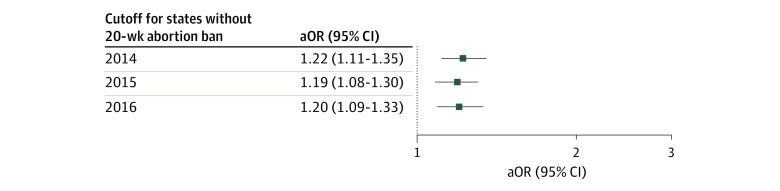
Association Between States With Abortion Ban Enactment and Down Syndrome After Ban Enactment, Using 3 Different Arbitrary Cutoff Dates for the States Without Abortion Ban Rates of Down syndrome increased in states with a 20-week abortion ban after the enactment of the ban. All models controlled for state, year of birth, maternal race and ethnicity, maternal age, educational level, insurance status, and number of prenatal visits. aOR indicates adjusted odds ratio.

On multivariable logistic regression assessing the interaction of time and group (states with vs without 20-week abortion ban), the odds of Down syndrome were higher in states that enacted 20-week abortion bans after the law enactment compared with the years prior to ban enactment (adjusted odds ratio [aOR], 1.22; 95% CI, 1.11-1.35), using 2014 as cutoff for the states without a 20-week abortion ban (Figure 3). Sensitivity analyses with a cutoff of 2015 (aOR, 1.19; 95% CI, 1.08-1.30) and 2016 (aOR, 1.20; 95% CI, 1.09-1.33) showed similar results.

## Discussion

In our study, we demonstrated that, in the US between 2011 and 2018, the odds of a neonatal Down syndrome diagnosis were higher in states that enacted 20-week abortion bans in this period compared with states that did not enact such bans. Although states with and states without bans had statistically insignificant differences in neonates who received a diagnosis of Down syndrome in 2011, by 2018 there were more diagnoses in states with 20-week abortion bans. Although demographic and individual characteristics differed between states with and states without 20-week bans, when adjusting for cofounders as well as state, year of ban enactment, and year of birth, deliveries in states with bans were more likely to be associated with a neonatal diagnosis of Down syndrome than those in states without bans. From our multivariable analysis, we found that births in states that enacted 20-week abortion bans were 1.22 times as likely to have a diagnosis of Down syndrome than those in states without 20-week bans. Assuming a baseline birth prevalence of 1 in 700, or 6000 births with a diagnosis of Down syndrome per year, a national 20-week abortion ban would be associated with an increase of 1320 cases annually.

Our results demonstrate a difference in the change in birth prevalence of neonatal Down syndrome between states with and states without abortion bans in our study period. Although it is possible that there are multiple causes for changes in the rates of diagnosis among this large population cohort, our results persisted when adjusting for confounders and year of ban. Because 20-week abortion bans are known to prevent the option of termination in the setting of prenatal diagnoses that occur during second-trimester screening, it is possible that the shifts we note were due in part to this changing legislation.^3,17^

### Limitations

Although the results of our study are consistent with current literature, our study is not without limitations. Some data show that there is low sensitivity for Down syndrome diagnosis in birth certificate data and that the sensitivity of these data may vary by factors such as maternal age, race and ethnicity, educational level, preterm birth, and hospital size.^24,25^ However, the goal of this study was to compare differences in rates of Down syndrome diagnosis over time between states that enacted 20-week abortion bans and those that did not. Therefore, we are not seeking to capture or evaluate all cases of Down syndrome. If the low sensitivity of Down syndrome is relatively consistent over all the data by additional factors that we have included in our multivariable analysis, then the association of abortion restrictions with Down syndrome diagnoses may still be identified.

As with any large, historical, population-based study, it is impossible to determine causality with respect to abortion legislature. Because each state enacted a ban along a different timeline, it is impossible to distinguish exact patterns in increases in diagnoses, especially regarding when new legislation would begin to affect clinical practice in this context. Furthermore, while we were able to control for many variables in our analysis, there were some confounders we were unable to address due to the constraints of the data. For instance, we could not address differences in clinical practice, including type of aneuploidy screening, that occurred for each pregnancy, which may have differed by state and by year. It is possible that, as noninvasive prenatal screening became more highly used over the decade, more terminations prior to 20 weeks would have occurred. However, this would likely have affected both states with and states without 20-week abortion bans, which allowed us to control for some of these changes in practice. Furthermore, our results are robust because our multivariable analysis found consistent results even when controlling for state and year of ban.

## Conclusions

This cohort study found that among births in states where 20-week abortion bans were enacted, there were increased odds of neonatal diagnosis of Down syndrome compared with states without bans. These results suggest that 20-week abortion bans may inhibit choice in decision-making in the context of second-trimester aneuploidy screening. Terminating a pregnancy is a highly individual, personal decision between pregnant patients and their clinicians. Physicians and politicians should advocate against such bans to allow all pregnant individuals in the US to make informed decisions, regardless of state of residence.

## Notes

Supplement.
Data Sharing Statement
