# Supplementary material for: Association Between Rates of Down Syndrome Diagnosis in States With vs Without 20-Week Abortion Bans From 2011 to 2018
Source: JAMA Netw Open. 2023 Mar 21;6(3):e233684. doi: 10.1001/jamanetworkopen.2023.3684 (PMC10031387; doi:10.1001/jamanetworkopen.2023.3684)
Supplement: Supplement. — Data Sharing Statement [file jamanetwopen-e233684-s001.pdf]

# Data Sharing Statement

Chaiken. Association Between Rates of Down Syndrome Diagnosis in States With vs Without 20-Week Abortion Bans From 2011 to 2018. *JAMA Netw Open*. Published March 21, 2023. doi:10.1001/jamanetworkopen.2023.3684

**Data**
**Data available:** No

**Additional Information**
**Explanation for why data not available:** We will not be sharing the data as this dataset is publicly available.
